# Should states restrict recipient choice amongst relevant and available COVID-19 vaccines?

**DOI:** 10.1093/medlaw/fwac042

**Published:** 2022-10-14

**Authors:** Emma Cave, Aisling McMahon

**Affiliations:** Durham Law School, Durham University, Durham, England; Department of Law, Maynooth University, Maynooth, Ireland

**Keywords:** COVID-19, Human rights, Informed consent, Patient choice, Vaccination, Vaccine equity

## Abstract

Several COVID-19 vaccinations have been authorised worldwide. Whilst some vaccines are contraindicated for certain age groups or health conditions, there are often multiple clinically suitable authorised vaccine brands available. Few states have allowed recipients to choose amongst them, though there are multiple reasons why choice would be valued. We consider the policy justifications for state controls on recipient choice amongst COVID-19 vaccine brands, focusing on European countries and drawing on the UK context as an example. We contrast justifications for not offering choice at the height of the early pandemic crisis, and as some states seek to de-escalate their response and transition towards living with COVID-19. We argue that in the latter context public expectations of choice between available vaccine brands and platforms may rise, but that several considerations may justify continued restrictions on choice. A key factor which states should continue to take into consideration is the global nature of the pandemic. Insofar as offering recipient choice at a national level might exacerbate global inequity in vaccine distribution, states retain a normative and legal justification for restricting choice amongst available and clinically suitable vaccine brands.

## I. INTRODUCTION

The public health emergency that followed the emergence and spread of the highly transmissible, high mortality and morbidity SARS-CoV-2 (COVID-19) virus has resulted in multiple vaccine candidates. In summer 2022, the World Health Organisation (WHO) listed 170 vaccines in clinical development and a further 198 in pre-clinical development.^1^ Funding, collaboration and reduced bureaucracy led to several fast-tracked vaccines being authorised for use in humans.^2^ By August 2022, over 69% of the world population have received at least one dose of a COVID-19 vaccine and 2.3 billion booster doses have been administered.^3^

The UK was the first country to approve a COVID-19 vaccine that has been tested in a large-scale clinical trial.^4^ The Medicines and Healthcare products Regulatory Agency (MHRA) gave temporary authorisation for adult use of batches of the Pfizer BioNTech (or Comirnaty) vaccine in December 2020 and later extended temporary authorisation to children from age five; The AstraZeneca vaccine (Vaxzevria) was authorised in December 2020; Spikevax/Moderna was authorised for adult use in January 2021 and later extended to children aged twelve to seventeen in August 2021; and Janssen/Johnson & Johnson was approved in May 2021 and requires one dose. The Janssen vaccine has not been administered in the UK and ordered vaccines were donated to developing nations.^5^ The other three COVID-19 vaccines currently require a two-dose initial course. From autumn 2021 the UK in common with many other countries recommended a booster regimen (a third dose) for adults,^6^ and certain high risk/vulnerable groups could obtain a third dose and booster (fourth dose).^7^ Since then, Nuvaxovid (Novavax) was given conditional authorisation for use in the UK in February 2022,^8^ Valneva in April 2022,^9^ and a Spikevax/Moderna bivalent booster vaccine designed to tackle both the original virus and the Omicron variant in August 2022.^10^ The UK Joint Committee on Vaccination and Immunisation (JCVI)^11^ has issued advice on priority groups, vaccine intervals, contraindications and age categories which, at the discretion of the state,^12^ limit the clinical relevance of some of the vaccines to certain populations. The core six vaccines have been conditionally authorised for use in the EU where, in August 2022, five more vaccines are under consideration.^13^

At the time of writing, in summer 2022, COVID-19 is still capable of causing devastation—it can prove fatal for some people, there are risks of Long COVID-19 and a new variant could emerge at any time which may be resistant to existing vaccines. Nonetheless, deaths are declining, and there is evidence that COVID-19 could eventually become endemic.^14^ Booster campaigns will be relevant as immunity wanes and adaptations and new vaccines may be required to tackle new variants of concern. Plus, given the number of vaccines under development,^15^ efficacy and safety may be improved in time. Thus, since spring 2020, for some cohorts at least, there has been a range of alternative vaccines against COVID-19 temporarily authorised for use and the multiplicity of vaccines is likely to endure for some time to come. Yet, though most European countries have recommended conditional authorisation for more than one COVID-19 vaccine, Serbia was reportedly the only European country where people were able to choose which vaccine they wanted.^16^ Intuitively, this might seem to contrast with the position with regard to medical treatment, where availability of more than one clinically relevant treatment alternative will often lead to the state allowing the choice amongst them to be driven by a patient’s values and preferences.^17^ In this article, we ask: If there is no valid clinical reason for a vaccinator to select vaccine A over B or C, and all three are authorised by the state and available, when, if at all, should states allow recipients to choose amongst them?^18^ Our focus is on policy in Europe and at various points we refer to the UK position as an illustrative case in point.

In focusing on this question, we acknowledge differences between medical treatments and vaccines—in particular, vaccines adopted within a broader public health context give rise to peculiar considerations, especially for contagious diseases—a point we develop further below. We also acknowledge that COVID-19 is atypical in that there are several different types of vaccine brands and platforms available where many were developed concurrently. This is not generally the case within the vaccine context. Indeed, for many conditions there are limited incentives for the market to produce new vaccines—vaccine research and development is commonly recognised as an area of market failure. Thus, the scenario of having multiple different vaccines for a virus is not a common one. Nonetheless, for COVID-19 and future pandemics, which may give rise to a similar ‘race to vaccines’ given high global demand and assuming the private market model is retained, it is important to consider how patient choice over available approved vaccines should be balanced against other relevant factors in public health policy.

Giving individuals choice and control over healthcare outcomes can help to promote their human rights to privacy and self-determination. The European Court of Human Rights has recognised the intrinsic value of self-determination which is protected by Article 8(1) of the European Convention on Human Rights (ECHR).^19^ Article 8 protects the right to a private and family life, which extends to freedoms to accept or refuse medical treatment.^20^ However, whether Article 8 might also extend to protect rights to choose amongst available vaccine alternatives is currently unclear, and the arguments are discussed below.

We contrast the recipient’s right to choose amongst available vaccine brands in the ‘crisis’ situation when COVID-19 vaccines were first introduced, with emerging strategies in some, often high-income countries (HICs) that seek to de-escalate emergency measures and transition to ‘living with COVID’. We demonstrate that, in the latter context, some of the factors relevant to the justification of choice restriction for vaccine recipients in a crisis fall away and public expectations of choice may rise. Nonetheless, we argue that these factors must be balanced with the risks associated with offering choice given the dangers of new variants of concern, and that consideration should be given to the potential impact of enhanced recipient choice on global vaccine inequity. We consider whether restrictions on choice are compliant with the ECHR.

The article is in four parts. Part II considers the policy justifications for recipient choice restrictions when multiple vaccines were first made available in the early phase of the COVID-19 pandemic. We acknowledge the potential value of choice amongst available vaccines but consider the limits of a consumeristic conception of patient choice, particularly in an emergency pandemic situation. We explore the policy justifications for state restrictions on COVID-19 vaccine choice for recipients where authorised alternatives could be obtained.^21^ In particular, we contrast the benefits of self-determination and patient centred care in medical treatment, and utilitarian ethics in a public health crisis, where the priority shifts to protecting the physical health of the maximum number of people.

In Part III, we consider the potential for choice amongst vaccine brands to be highly valued as some HICs have sought to effect a partial return to normality: a relaxation of control measures, and distancing from perceptions of crisis management.^22^ In England, this period is marked by the ending of certain legal restrictions in late February 2022.^23^ From this point, as we shall see, public expectations of choice might rise as some of the justifications for choice restriction for vaccine recipients no longer apply.

In Part IV, we accept that any return to ‘normality’ in living with COVID-19 strategies must incorporate elements of adaptation to the long-term impacts of COVID-19 and the ‘normalisation’ of aspects of COVID-19 management.^24^ We focus in particular on the relevance of global equity. In spring 2022 as a policy of living with COVID became increasingly prevalent in Europe, there was significant disparity in access to vaccines between HICs and low-middle-income countries (LMICs).^25^ We argue that it is morally and pragmatically objectionable to offer recipients in a particular state vaccine choice if doing so maintains or exacerbates global inequity in vaccine roll out. We call for a recognition that the global levels of vaccination impact on the progress and threat of COVID-19 in particular states.^26^ We argue that the value of enhanced choice amongst COVID-19 vaccines to people should be measured against the potential impact of offering vaccine choice on the public health in the particular state and its global impact, and that this global picture must remain in the minds of those implementing national policies for COVID-19 and future pandemics. Provided there is a credible causal link between increased choice in a particular state and global inequity, there remains a powerful—moral and utilitarian—normative justification for choice restriction.

Despite this strong normative case, from a legal perspective an obstacle to accounting for global equity concerns at a national level in continuing to restrict recipient choice, is that it is plausible individuals may seek to challenge lack of choice amongst vaccines in HICs. Thus, in Part V, we consider the legal implications of restricting choice, focusing in particular on the likely human rights claims against a state policy to this effect that could arise in this context. We argue that the global nature of the pandemic is pertinent to the necessity and proportionality of restrictions on choice at a national level.

## II. VACCINE BRAND CHOICE IN A NATIONAL EMERGENCY

In this section, we consider the justifications for states restricting choice for recipients amongst vaccine brands in a national emergency context when vaccines were first rolled out. In the UK, this period commenced in December 2020.^27^

We start by recounting the potential policy rationales for offering recipients choice amongst vaccine brands where multiple clinically relevant options are authorised and available. One such advantage flows from the intrinsic value of choice to recipients because of perceived and actual variability of risks and benefits amongst vaccines. Examples include:

1. In the UK and EU authorisations for supply were given to vaccines using different platforms which carried different actual and perceived risks and benefits. Pfizer BioNTech/Comirnaty and Spikevax/Moderna utilise an mRNA platform which uses the vaccine’s genetic code to trigger the production of a protein in our bodies that matches the viral protein and stimulates an immune response. AstraZeneca/Vaxzevria and Janssen, on the other hand, are adenovirus vector vaccine which use a genetically engineered virus which enters cells in the body and produces a harmless piece of the COVID-19 virus, triggering an immune response. In early 2021, some might have preferred the mRNA vaccines given evidence of favourable efficacy.^28^ Conversely, mRNA vaccines are a relatively new type of vaccine which may have exacerbated public concerns over short and long-term effects, notwithstanding that studies indicate that the risks are similar to those of other viral vaccines.^29^ The novelty of mRNA vaccines might therefore have led some to have chosen the adenovirus vector had they been given the choice.^30^ Later in the pandemic, additional platforms were added: Nuvaxovid, approved in the UK in February 2022,^31^ is a protein subunit vaccine. Valneva, approved in April 2022,^32^ is a whole-virus vaccine where the virus is inactivated so that it cannot replicate but is still capable of triggering an immune response. These two vaccines rely on technologies used in flu, whooping cough, meningococcal infection, and polio vaccines, which might alleviate some of the concerns of those who are vaccine hesitant;
2. As risks emerged in relation to different age groups and risk factors, states differed in their responses. Take for example the data that emerged in spring 2021 about the small risk of a rare blood clot disorder in younger people receiving the AstraZeneca vaccine. In April 2021, the UK changed its advice around the AstraZeneca vaccine for people under the age of 30, but other countries imposed more extensive restrictions. Canada and France restricted the vaccine to those aged fifty-five and over and Germany and Iceland set an even higher age cut-off.^33^ A concerned 31-year-old in the UK might have valued the option of choosing another vaccine considering the policies adopted by other countries and the lack of global consensus as to the degree of risk;
3. The long-term risks and benefits associated with novel vaccines^34^ are also pertinent, and if offered choice recipients may have weighed relevant factors differently depending on their own subjective values, health and other circumstances. Consider the UK booster regime rolled out in autumn 2021 to improve immunity for those who had completed the two-dose course. Given the choice, some might have actively sought out different vaccine brands in a bid to expand their protection by increasing their antibodies through ‘pick and mix’ vaccination.^35^ Others might have wished to adhere to the same vaccine brand as their primary vaccine course to avoid exposure to the risks associated with two novel vaccines. In England, neither such choice was formally accommodated for recipients. The JCVI advised that because in trials mRNA vaccines showed the strongest impact on immunity, only if mRNA vaccines were contraindicated should the AstraZeneca vaccine be given in place of the preferred Pfizer of Spikevax, and even then, only for those who had originally received AstraZeneca^36^;
4. A more practical benefit of choice related to restrictions on international travel for those who had received certain vaccines or batches^37^;
5. Finally, where state guidance recommended particular vaccine/s that were in short supply, some might have judged the risks associated with taking an alternative vaccine to be worth the reduction in wait time. In Ireland, for example, people in their 40s were not recommended the AstraZeneca or Janssen vaccines but were told that they could access them subject to certain conditions if alternatives from Pfizer or Spikevax were unavailable.^38^

From a policy perspective, the value of choice to individuals can impact on public expectations, particularly in states with high levels of consumerism. Consumerism is relatively limited in a nationalised health system such as the UK, where services are provided based on need and are funded through taxation. But even in the UK, emphasis on rights and consumerism reduces the passivity of potential vaccine recipients. There is evidence that this has been exacerbated in the pandemic, fuelled by telehealth, remote consultations and increasing reliance on artificial intelligence.^39^ Consumerism was referred to and seemingly accepted in the health context by the Supreme Court in *Montgomery v Lanarkshire Health Board*^40^ (*Montgomery*) where Lords Kerr and Reed stated:

[P]atients are now widely regarded as persons holding rights, rather than as the passive recipients of the care of the medical profession. They are also widely treated as consumers exercising choices: a viewpoint which has underpinned some of the developments in the provision of healthcare services.^41^

Satisfying expectations of choice has been linked to enhanced user satisfaction and to good health outcomes.^42^ Patient expectations of choice are therefore a pertinent policy consideration in some contexts.

If states are not influenced by the intrinsic value of choice to individuals and the expectations this raises, they may be influenced by its instrumental value. Vaccine hesitancy is variable and volatile and has been problematic in some European states.^43^ Even within states with good uptake, structural inequalities make some populations hard to reach.^44^ In both cases, agile vaccination strategies are important and choice is a possible mitigation tool. Additionally, some people expressed moral concerns in relation to some vaccines, particularly, for example, if research had relied upon cell lines historically linked to abortion. Putting aside for the moment the probity of such views,^45^ offering recipients a choice of vaccines might have limited vaccine refusal. In the UK, for example, two brands of the measles, mumps, rubella (MMR) vaccine are available: MMRVaxPro and Priorix. Priorix does not contain porcine gelatine and can be offered as an alternative to MMRVaxPro for those who object to the porcine content for religious or moral reasons.^46^ Sprengholz and others have demonstrated in a choice experiment that responding to COVID-19 vaccination preferences can improve uptake.^47^ Indeed, in Serbia, where the government promoted choice of COVID-19 vaccines, rates were the second highest in Europe in February 2021 before a subsequent downturn fuelled by vaccine hesitancy.^48^ Hughes and others have argued that choice amongst vaccines may mitigate health inequalities and reduce vaccine hesitancy in a national context ‘by showing people that they are not being forced into a “take it or leave it” decision’.^49^ Gavi, a vaccine partnership alliance, has argued that a recognised benefit of Novavax is that:

Expanding the choice of vaccines, particularly those based on an established technology with a good safety record, could … help drive vaccine acceptance and boost the fight against the pandemic.^50^

An additional consideration for countries that issued mandates to improve COVID-19 vaccine uptake, is that choice amongst vaccines might have enhanced compliance with Article 8 of the ECHR. In the case of routine childhood (non-COVID-19) vaccination, the European Court of Human Right in *Vavřička and Others v the Czech Republic*^51^ recognised that where a parent refuses to provide a valid consent, a state can impose proportionate and necessary penalties without breaching the Convention.^52^ Whilst it would be naïve to assume that choice amongst available vaccines would appease those opposed to a vaccine mandate, it does at least have the potential to render mandated vaccination more proportionate, and thus more likely to be justified under Article 8(2) of the ECHR.

There is therefore reason to take seriously the potential benefits of offering choice to individual recipients of available COVID-19 vaccines at a national level, but these factors must be weighed against utilitarian counterarguments in the pandemic context.^53^ The scale of the global health emergency presented by COVID-19, the lack of effective treatment options for COVID-19^54^ and the costs of enforced social distancing made a speedy vaccine strategy the most effective means of containing COVID-19 and of the lifting of public health restrictions, in as quick a timeframe as possible. In the UK, this factor was expressly conveyed by the JCVI in its 2020 guidance on priority groups, stating that: ‘For operational and programmatic reasons, such as to enable more extensive and timely vaccine coverage, one vaccine may be offered in certain settings in preference over another vaccine.’^55^ This position was bolstered by the fact that at that point in the pandemic, no clinical trial had compared Pfizer and AstraZeneca vaccines (the main vaccines then approved), and so a preference for either in any specific population could not be justified.^56^ This strategy offered the highest chances of reducing the spread of severe illness, limiting the overall loss of life in a population, and reducing the burden of care which high numbers of people suffering from COVID-19 in unvaccinated populations present. Furthermore, maximising the numbers vaccinated in a country can reduce indirect loss of life related to the pandemic—in this context, particularly, during the early stages of the pandemic, healthcare settings in many countries including the UK^57^ had to pause other healthcare services due to the burden of care presented by COVID-19 and risks of transmissibility of COVID-19 to vulnerable groups within healthcare settings. Limiting other healthcare services resulted in indirect deaths, and poorer health outcomes such as those arising from undiagnosed or untreated cancers and other illnesses.^58^

Even assuming adequate supply of several approved COVID-19 vaccines, offering a choice of vaccines early in the pandemic could conceivably have delayed the vaccination drive in the UK and other countries, in three main ways:

1. logistically it may have required greater training of medical personnel administering and providing information on different types of vaccines. In the UK, an expanded workforce was created which collectively had less experience of vaccine administration.^59^ At a bureaucratic level, the different types of vaccines administered to each person if choice was available would all need to be recorded. And either storage and facilities for different vaccines in the same vaccine centre would be required, or multiple sites would be needed to administer different vaccines. Although different vaccines were offered in some cases to different age cohorts, this is still logistically more straightforward as the state was controlling who gained access based on age or comorbidity, not based on recipients’ choice, and so it would likely be easier to determine the numbers of each cohort who would require specific vaccines, and to record who gained access to which vaccine;
2. offering a choice of vaccine could potentially have led to confusion for people over which vaccines they should take which may have delayed their uptake of vaccines. This factor may have been exacerbated in the early stages of vaccination rollout when vaccines were new. Whilst we often value choice, it can also bring dilemmas, confusion and difficulty, particularly when information around a healthcare intervention is limited by a product’s novelty;
3. some vaccines may have been favoured by people within a state, which could lead to shortfalls in supply of preferred vaccines, and surpluses of other vaccines. Even in the absence of offering a choice of vaccines *per se*, there is some evidence that people may have delayed or refused vaccination to try to obtain their preferred COVID-19 vaccine at a later stage.^60^

Relatedly, supply issues for COVID-19 vaccines meant that states did not (and some—particularly LMICs—still do not^61^) as will be discussed below, have adequate supplies of approved vaccines to meet population needs,^62^ nor did they have equal supplies of alternative vaccines that may have been approved in that state. Even in HICs where COVID-19 vaccine alternatives were more readily available, offering a choice of vaccines could have delayed the overall vaccination campaign because short supply of a more popular vaccine would have caused delay even though vaccines with a similar risk and benefit profile were available.

These factors are relevant to policy makers, but they also impact on the value of choice to individuals. It is important that we critically reflect on how patient autonomy and the role of healthcare providers are conceived of in modern medical law,^63^ and reflect on the likelihood of whether offering vaccine choice early in the pandemic would have met recipients’ needs at a population level. There are aspects of consumeristic choice that are problematic in relation to treatment as well as vaccination, and in both pandemic and non-pandemic situations. It has been argued that consumerism can lead to unmet expectations when in fact choices are limited by factors such as limited resources, evidence of clinical need and requirements not to cause harm.^64^ Thus, from a public policy perspective a public expectation of choice in healthcare is something to be managed in order to focus on what can realistically and helpfully be achieved. Furthermore, the consumeristic conception of rights has been criticised as being overly individualistic and detached from the empirical realities of patients’ actual needs.^65^ Many patients do not view their relationship with healthcare providers in consumerist terms, and empirical work shows some patients who trust medical professionals will prefer to defer to them on at least some aspects of healthcare decisions.^66^ Empirical research shows that particularly where patients are faced with difficult decisions they may find it emotionally difficult to process information at consultations,^67^ and that such issues are exacerbated when a patient is vulnerable.^68^

Applying this in the context of the early stages of a global pandemic caused by a novel virus, when many people are likely to feel heightened anxiety for their health, a universal desire for choice amongst available COVID-19 vaccine brands—many of which were novel vaccines—should not be assumed. Some people may prefer to defer to medical expertise even if a choice were possible (which it often was not in the COVID-19 crisis given the limited supplies of vaccines at that time even in HICs). Moreover, early in the pandemic, a focus on choice provision alone was unlikely to reflect patients’ empirical needs, unless it could be embedded within a shared decision-making model of healthcare which maintains a role for healthcare providers’ expertise in facilitating patients to make decisions to suit their needs and values, whereby choices would be made by patients in dialogue with healthcare providers.^69^ Facilitating effective shared decision-making is dependent upon resources, including additional medical staff and time to discuss options available^70^ that were in short supply, particularly as healthcare workers were needed to deal with surges of COVID-19. Thus, pragmatically, the value of choice when vaccines were first authorised has to be questioned. Moreover, this was particularly so when the variable difference between vaccines was either unknown or where known, minimal. In these circumstances, it is likely that offering choice amongst approved vaccines would not be desired by all, and where it was desired, many individuals were unlikely at that point in the pandemic to be equipped with the tools and support needed to make an informed choice amongst vaccines.

In conclusion, early in the pandemic, the value of autonomy to recipients was a relevant policy consideration, but it was balanced against the limitations of its intrinsic value in a pandemic context, particularly given the multiple variables and shifting evidence which made it difficult for individuals to judge which vaccine may be ‘best’ for them.^71^ The evidence suggested that having *any* of the vaccines approved for that patient population would offer strong protection against COVID-19, low risk of adverse events and was thus vastly superior to the risks associated with contracting COVID-19.^72^ Moreover, providing choice, would have had limited instrumental value given high uptake in most countries where vaccines were made available at that stage in the pandemic, and several utilitarian considerations have operated against providing choice amongst brands. The potential for delays associated with increased choice at the recipient level and the public health imperative to reach maximum numbers with maximum speed are chief amongst them. Considering this, we would argue that the state’s population level decision to limit recipients’ choice of vaccines from early in the pandemic was normatively justified.

## III. CHOICE IN COUNTRIES TRANSITIONING TOWARDS ‘NORMALITY’

However, in HICs from spring 2022, the balance between individual autonomy and public health goals changed as the approach to COVID-19 moved away from urgent crisis management and elements of ‘normalcy’ were returned under so-called ‘living with COVID-19’ strategies. In the UK, the Coronavirus Act 2020 conferred powers and new temporary flexibilities on many policy areas, at a time of national emergency. It was passed speedily and renewed every 6 months. As in many countries, the public health restrictive measures relied on broad public acceptance and compliance, which can wane the longer they continue and the further the state is from national healthcare system collapse.^73^ In February 2022, many COVID-19 legal restrictions in England were lifted.^74^ Though the pandemic was not over, and ongoing threats of new variants remained, a new phase of ‘living with COVID’ was announced.^75^ The Government referenced the successful vaccination campaign that led to the UK being the first major European economy to provide booster vaccinations to more than 50% of the population.^76^ Those—usually HICs—that have initiated successful booster campaigns found themselves in a position to begin scaling back emergency COVID-19 legislation.

The lifting of restrictions in England came notwithstanding a warning from the UK’s Chief Medical Officer, Chris Whitty, that future COVID-19 variants could be more dangerous and more resistant to existing vaccines than the Omicron variant,^77^ and could even potentially require new vaccines to be developed. Thus from spring 2022, there remained and remains at the time of writing a possibility that re-escalation could be required, in which case the arguments set out in Part II justifying choice restriction would regain force, particularly if a new variant arose and new vaccines were needed to address it. In that scenario, if our current vaccine production model is not changed, global shortages of new vaccines needed to meet global demands are likely, and a reversion towards emergency public health goals will be needed. Thus, vaccine development, adaptation and deployment will remain a policy priority for as long as the risk of new COVID-19 variants of concern remains. Similar issues are also likely if other pandemics arise.^78^ But in the COVID-19 context, pending the emergence of another serious COVID-19 variant, de-escalation in HICs is likely to lead to regular booster campaigns, and this will also impact on the justifiability of restricting recipients’ choice around COVID-19 vaccines in HICs.

It is important to note that, even in a transition to endemic conditions, there are ongoing risks to the public health that might justify restrictions on choice to aid efficiency and reduce costs. A disease can be both endemic and high risk and waves of endemic infection can be highly disruptive.^79^ Nevertheless, de-escalation from pandemic emergency could impact in several ways on the arguments set out in Part II justifying recipient choice restriction across available COVID-19 vaccines. In a de-escalation scenario, vaccine supplies are often stabilised, particularly in HICs. Choices amongst which vaccines to take that may have confused individuals early in the pandemic, are potentially easier given the time that has elapsed since COVID-19 vaccines were first administered resulting in increased quality and quantity of published research and the improved availability of healthcare professionals to provide pertinent information to aid choice. There may be increased instrumental justification to increase choice if those whose initial scepticism was directed at a vaccine rather than all COVID-19 vaccines, might be encouraged to take-up vaccination.^80^ Public expectations, which are one of a number of factors pertinent to public health policy,^81^ may also change as the paradigm shifts away from a public health emergency and vaccinations against COVID-19 become more routine.

The latter factor could be influenced by relatively greater alignment of vaccination with treatment outside of a crisis scenario. Whilst most patients will recognise key differences between preventative vaccination and proactive treatment; public health focussed measures and personal treatment options, de-escalation of the crisis has potential to bring the two medical interventions into closer alignment and impact on patient expectations. This could conceivably flow from the move away from vaccinations taking place in temporary vaccination centres in favour of traditional healthcare settings such as GP surgeries which were historically focussed on reactive healthcare, but now extend to health promotion and preventative medicine such as routine vaccination. Additionally, the bright line between proactive and reactive healthcare is blurred by the development and deployment of vaccines that are reactive to new and prevalent strains, as we see in relation to annual influenza programmes. Such a transition might also be influenced by the merging of vaccination and treatment in aspects of the law. For example, statute and case law in England and Wales suggests that no clear distinction can be drawn between treatment and vaccination. For the purposes of Part 2A section 45E of the Public Heath (Control of Disease) Act 1984, which concerns public health protection, medical treatment incorporates vaccination. And King LJ in the Court of Appeal decision of *Re H*^82^ recognised that medical treatment and preventative healthcare are overlapping concepts. We can hypothesise then that the differences between the pandemic emergency scenario and a de-escalated ‘living with COVID-19’ scenario, coupled with the unusual availability and clinical relevance of multiple vaccine brands and the potential for recipient preferences amongst them as set out in Part II, might exacerbate public expectations of choice.

The principles of self-determination and choice that have guided recent changes of the law on choices between available treatment alternatives and variants in the landmark negligence case of *Montgomery*^83^ were based on a series of social changes that are also relevant to patient choice amongst vaccine alternatives. The principles include doctor–patient partnership,^84^ support,^85^ self-determination,^86^ and crucially, a shift away from paternalism^87^ to support a rights-based conception of patient choice. The expectations relevant to patient choice in *Montgomery* in the UK—and similar trends can also be observed in other European states^88^—might increasingly become pertinent to vaccine recipient expectations of choice, notwithstanding that vaccination is preventative and public health focussed rather than reactive and treatment focussed.

In this section we have hypothesised that de-escalation could lead to pressure in some countries to extend choice between available and relevant vaccine brands. Public health is by nature highly political, making public expectations of choice an important consideration. It is a consideration that must be balanced with the benefits of choice restriction in a potential resurgence of pandemic emergency conditions, epidemic conditions and in endemic COVID-19 conditions. Our focus in this section has been on inward looking national policy considerations. In the next section we consider the importance of turning outwards to consider the global impact of national strategies around recipient choice.

## IV. NATIONAL CONSIDERATIONS OF GLOBAL INEQUITY

In reviewing policies on recipient choice amongst COVID-19 vaccines, we argue in this section that states should be mindful of the potential impact of national policies on global vaccine roll-out. In winter 2021 the payment of higher prices for boosters in HICs than first/second doses in LMICs, resulted in vaccine doses being diverted to HICs.^89^ In December 2021 the *Financial Times* reported that ‘boosters in rich countries outnumber all jabs among poor nations’.^90^ By April 2022, 71.9% of people in HICs had been vaccinated with one dose or more of a COVID-19 vaccine, compared to only 15.21% of people in low income countries.^91^ For example in the UK, 74% of people had received two doses and 59% had received a booster, whereas in Ethiopia only 22% had received a first dose.^92^ The analogy used by Dr Mike Ryan (WHO) to describe this is that it is akin to providing an additional life jacket to some, leaving others without any to drown^93^—arguably exacerbated by the fact that many LMICs have health care systems which are less well functioning than those of HICs, and are most likely to struggle with providing care to those suffering with COVID-19.^94^ Yet given the discussions around the Omicron variant and the protection offered by booster vaccines, it is understandable that at the national level HICs came under pressure to offer booster doses to protect national populations. Much has been written on the ethical issues against^95^ and in support^96^ of ‘vaccine nationalism’ in HICs—which can generally be defined as policies adopted in HICs which prioritise access to vaccines for those within their state boundaries even where policies such as stockpiling of vaccine doses beyond their population needs, are contradictory to needs of other populations. We do not seek to repeat those arguments here. We do, however, raise the relevance of the global landscape in considering justifications for choice restriction at a state level, if there is evidence that choice in one state would potentially exacerbate global inequity.

In this context, a strong moral and pragmatic/utilitarian rationale for limiting vaccine choice in the UK, has been the significant global inequity in relation to COVID-19 vaccine access between HICs and LMICs. Arguably, if HICs were, in the context of global vaccine inequity, to offer a choice amongst vaccine brands, at a point in time where there was limited availability of vaccines in many other (primarily, LMICs), this could have further entrenched the differences between those in HICs and LMICs, and from a moral perspective would have been highly problematic.

If a state were to offer recipient choice between COVID-19 vaccines, it is difficult to predict which brand recipients would choose, as we have seen when exploring the various reasons why one vaccine might be preferred over another in Part II. As a result, states would need to stockpile sufficient alternatives to allow choice, and this could in turn plausibly impact upon supplies available in LMICs.^97^ This is because many LMICs have traditionally had limited capacity to manufacture their own vaccine supplies for various reasons, including due to applicable intellectual property rights and lack of technology transfer which we explore briefly below. This has made many LMICs reliant on donations from HICs^98^ to access any COVID-19 vaccine, vaccines with a workable shelf-life,^99^ and to facilitate selection at state level between the safest and most effective vaccines to administer to particular cohorts or as protection against a particular variant. Supply is far from the only issue that has impacted on inequity. There are, for example, important issues around the need for greater financing and support in building health system capacity for vaccine delivery, storage and distribution in LMICs and, as we discussed above, there are some issues around vaccine hesitancy.^100^ Nonetheless, and notwithstanding initiatives to procure COVID-19 vaccines in an equitable manner,^101^ a key issue for much of the pandemic has been a lack of supply of effective and suitable vaccines for LMICs.^102^ Lord Boateng in a 2021 House of Lords debate reported that ‘low-income countries cannot expect to have widespread vaccine access before late 2023’.^103^

Additionally, from a practical self-interested and utilitarian perspective, at an epidemiological level, the risk that offering vaccine choice in HICs could exacerbate vaccine supply problems for LMICs means that prioritising vaccine choice for some states over global equity is not epidemiologically sound. From a scientific perspective, eliminating global vaccine inequity, and offering vaccination to as much of the *global* population as possible, offers the best protection for people in every state. Thus, there is not only a significant moral imperative to achieve global vaccine equity -and arguably also a significant moral issue with offering a choice of vaccines for some in relation to first, second and additional vaccine doses, by virtue of where they live, whilst others have no vaccines—but also a pragmatic and self-interested rationale for this. If any one country or region has a high number of their population that are unvaccinated, this runs a higher risk of acting as a reservoir for the virus in that state and leading to new variants of COVID-19 emerging which may be resistant to current vaccines. This in turn threatens the control of COVID-19 in every state. Leaving some populations without vaccines also threatens fresh outbreaks of the virus re-emerging in other states through travel from that state. Both concerns have sadly been raised in relation to the Omicron variant. As Dr Tedros Adhanom Ghebreyesus (Director-General, WHO) aptly stated:

The inequitable distribution of vaccines is not just a moral outrage, it is also economically and epidemiologically self-defeating.

The more transmission, the more variants. And the more variants that emerge, the more likely it is that they could evade vaccines.

And as long as the virus is circulating anywhere, the longer the global recovery will take.^104^

Both the moral and pragmatic concerns set out in this section relate to the potential impact of choice in some states on vaccine supplies in others. In making this argument, we do not suggest that restriction on recipient choice can address global inequity. Indeed, maintaining limits on choice will do little to address global inequity if additional action is not taken. We also stress that having to choose between providing recipient choice and providing global vaccine equity is not an inevitable choice—there is potential to achieve global equity *and* to offer recipient choice amongst vaccine brands. In particular, current and future supply issues could be ameliorated by upscaling production in LMICs for such vaccines. Since the start of the pandemic, there have been several proposed global mechanisms to increase the supply and speed of supply of vaccines globally,^105^ including the WHO’s COVID-19 Technology Access Pool (CTAP) which encourages rightsholders in the spirit of solidarity to share intellectual property (IP) rights, data, know how, and technology transfer around COVID-19 vaccines and other health technologies, to scale up the supply globally. Yet to date, no vaccine rightsholder has agreed to share such data, rights and knowledge for COVID-19 vaccines.^106^ Given the failure of such global mechanisms, and mounting vaccine inequity, in October 2020, India and South Africa proposed a temporary waiver of certain IP obligations under the Agreement on Trade-Related Aspects of IP Rights (TRIPS). If adopted, it would suspend IP rights for certain COVID-19 health technologies including vaccines. However, this proposal was opposed by many HICs and regions, including the UK and Europe.^107^ A compromised version of the waiver proposal –which is significantly narrower than the original proposal, with questionable usefulness in the vaccine context– was considered^108^ and adopted in June 2022.^109^ It remains to be seen to what extent this proposal will assist in increased production of COVID-19 vaccines, particularly, in the event of new vaccines being required if new variants of concern arise. In our view, the arguments raised above around the value in providing choice for people everywhere around which vaccines they can and should take, provide additional reasons why all states and particularly HICs should be adopting and encouraging other states to adopt additional measures to facilitate rapid upscaling of vaccines in LMICs and specifically, to facilitate global access to and uptake of the most relevant and appropriate vaccines for the cohort and virus strain.

The argument that limiting choice amongst vaccines is not a panacea for global vaccine inequity does not detract from its potential relevance as a factor in favour of a national state policy restricting choice amongst COVID-19 vaccine brands. Thus, if there is even a small risk that providing choice in some states would exacerbate lack of access to relevant vaccines elsewhere, from moral and epidemiological perspectives, it would be preferable not to provide choice than to do so at the risk of increasing (or maintaining) the likelihood of countries having large numbers of unvaccinated populations which will exacerbate inequality and offer ideal environments for new variants to emerge. It is in all our interests to increase vaccination efforts everywhere, if we are to control COVID-19 globally. As such, in relation to recipient choice between vaccine brands, as with other important aspects of pandemic policy, states seeking to normalise their response to COVID-19 must adapt to the new normal in preference to attempting to reassert the old normal.

Cumulatively, the potential for global inequity to be exacerbated by increased choice, and the national interest in reducing inequity provides an important policy consideration for states considering when or whether to introduce greater choice amongst COVID-19 vaccines. In spring 2022, as many HICs sought to de-escalate their COVID-19 responses, global inequity provided moral, practical, and related epidemiological justifications in states with sufficient supplies of alternative vaccines to limit recipient choice between vaccines. As global supply issues are gradually alleviated through worldwide relief efforts,^110^ the WHO has warned that it is ‘crucial to sustain and enhance the momentum for vaccination’ in light of reduced perceptions of risk, lower demand for vaccines, changing political priorities and continued risk of new waves and variants of concern.^111^

## V. HUMAN RIGHTS CONSIDERATIONS

So far, our focus has been on the justifiability of policy restrictions on recipient choice across COVID-19 vaccine brands, arguing that notwithstanding powerful arguments relating to the intrinsic and instrumental value of choice, the policies were justified and well tolerated at the height of the pandemic. We have hypothesised that in states that de-escalate COVID-19-related restrictions, there could be a corresponding increased expectation of vaccine choice, but argued that factors in favour of choice must be balanced with negative impacts on the public health, and with the impact of choice in any one country on global inequity. In this section we consider possible legal obligations to offer choice in European states that are de-escalating restrictions. Mechanisms used to protect human rights vary from one legal system to another. In the UK, for example, decisions of bodies exercising public functions can be challenged by way of judicial review at common law and on the obligations under the ECHR.^112^ We focus on ECHR human rights obligations which inform the operation of national legal remedies in Member States of the Council of Europe. Soft law from UNESCO^113^ is sometimes called upon to guide the European Court of Human Rights,^114^ and similarly, we will draw upon its principles.

Articles 2, 3, 8, and 9 have potential relevance in this context. Of these, Articles 2 and 3 ECHR are unlikely to aid an individual seeking to assert the right to choice across COVID-19 vaccine brands. Claims invoking the Article 2 right to life might be successful if medical care is inadequate and it leads to a person’s death.^115^ And Article 3’s prohibition on inhuman or degrading treatment is relevant if harmful treatment is imposed on adults with capacity against their will.^116^ However, neither such provisions are likely to apply in the context which we focus on here—ie in the context of a policy applicable in a state where vaccines are not mandatory, and where a person’s complaint relates to non-availability of choice amongst approved vaccines.

We saw above that some advocates for choice are motivated by perceived moral considerations relating to certain vaccine brands. An applicant might claim violation of Article 9 if the only COVID-19 vaccine offered offends their ethical or religious views, where alternatives that would not be considered offensive to the applicant are available to the state but denied to the applicant. Article 9 has two parts. Article 9(1) is not relevant in this context. It guarantees ‘freedom … in public or private, to manifest [one’s] religion or belief’ and cannot be interfered with by public authorities. Article 9(2), on the other hand, has potential relevance, however, it can be limited in order to protect (*inter alia*) public safety, health and the rights and freedoms of others. It protects the right to manifest religion in ‘worship, teaching, practice and observance’.^117^ States have a margin of appreciation as to when restrictions are necessary and legitimate that are likely to encompass public health decisions to restrict choice in order to plan for future spikes or new variants or to advance global vaccine equity.^118^

We would opine that a claim by an individual seeking choice between brands based on Article 8 is also, at the time of writing, unlikely to succeed, given our arguments above for public health justifications for choice limitation. Article 8 is perhaps the most open-ended of Convention rights, and Strasbourg Court jurisprudence has adapted over time to social and cultural change. Though there is not a case in point on the issue of choice between vaccine brands, Article 8 has increased potential relevance as public health justifications for choice limitation wane. The relevant parts of this qualified right read:

1. Everyone has the right to respect for his private … life …
2. There shall be no interference by a public authority with the exercise of this right except such as is in accordance with the law and is necessary in a democratic society in the interests of national security, public safety or the economic well-being of the country, for the prevention of disorder or crime, for the protection of health or morals, or for the protection of the rights and freedoms of others.

The aggrieved individual might seek to assert that the state arbitrarily interfered with Article 8, in which case if Article 8(1) is engaged, the interference is assessed in relation to Article 8(2). Alternatively, the applicant might allege that the state has breached a positive obligation in which case the failure (or partial failure) to act is considered on its merits. Thus, an applicant might claim that the state failed in its positive obligation to protect choice or that it interfered with the right to choice: either way, and notwithstanding the different types of obligation, the principles will apply in a similar manner.^119^

It is well established that respect for private life includes respect for physical, psychological or moral integrity.^120^ Whilst, in light of our arguments in Parts II and III, a lack of choice between vaccine brands might conceivably impact on physical, psychological and moral integrity, a fair balance is required between the individual’s interests and community interests.^121^ Each state has a ‘margin of appreciation’ on the applicability of Article 8, the breadth of which depends on the importance of the interest and how the state chooses to protect it.^122^ Purely technical disadvantages to the individual will not suffice to engage Article 8,^123^ though important points of principle are potentially relevant.^124^

On choice between vaccine brands, there is no directly applicable jurisprudence. In *Vilnes and Others v Norway*, Article 8 was violated when professional divers were not given sufficient information about risks to their health.^125^ An analogous argument might be made by prospective vaccine recipients that Article 8 is violated if, for example, states are insufficiently transparent about known and potential risks of certain vaccines on certain cohorts, so that people are unable to give informed consent to vaccination. In *Glass v UK*, the European Court of Human Rights acknowledged the right of patients to be involved in decisions made about their care.^126^ And the Court has made clear that a failure to fund a particular health treatment for reasons of resource allocation^127^ requires a ‘fair balance … to be struck between the general interest of the community and the interests of the individual’.^128^ But vaccine recipients offered vaccine A and refused the clinically viable vaccine B on the basis of global and national public health considerations, who are properly informed of the risks and who can choose not to have any vaccine will find it more difficult to demonstrate violation of Article 8.

In two narrow situations the prospect of success might be increased. Firstly, an applicant might seek to establish that the proffered vaccine is not in fact a clinically viable alternative. Imagine, for example, the following scenario: Fred has previously had two doses of Pfizer and presents at a pharmacy for a booster vaccination. He is offered Spikevax, notwithstanding plentiful supplies of Pfizer which the state has recommended as a potential booster vaccine. He would prefer Pfizer in order to avoid the theoretical long-term risks associated with exposure to two rather than one vaccine. Second, as we explored above in respect to Article 9, the individual might have moral concerns in relation to the assigned vaccine. If their request for an available alternative is refused, they might claim that their Article 8 rights are engaged and that the state has a positive obligation to offer them choice. In *Stoicescu v Romania*^129^ the applicant was attacked by a pack of dogs near her house in Bucharest and was seriously injured. The dogs, numbering around 200,000 in the city at the time, were widely regarded as a public health nuisance.^130^ She successfully argued that the state had failed in its Article 8 positive obligation to protect her ‘physical and psychological integrity’.^131^ An applicant might attempt to argue by analogy that a failure to offer choice of an alternative authorised and available brand similarly failed to protect their psychological integrity and potentially also their physical integrity if the assigned vaccine is later shown to be harmful and the applicant, if offered choice, would have avoided the risk of harm.

The balancing exercise required to show violation of Article 8 requires due consideration of the state interests in restricting choice. When vaccines were first introduced, there is a strong argument that interference was both necessary and proportionate, for the reasons we set out in Part II. As we discussed in Part III, in the context of de-escalation, an individual might argue that the matter is more finely balanced. If supplies of alternative vaccine brands are readily available, logistical issues resolved and information on alternatives (and risks profiles of various vaccines) is more reliable, then the expectation of and justification of choice are stronger. However, at the time of writing two counter-arguments apply. First, as we have seen, de-escalation usually denotes reduction but not elimination of the risks COVID-19 poses to the public health. A risk of resurgence remains possible, and even endemic COVID-19 is a serious and potentially disruptive factor that could justify the prioritisation of efficient and cost effective supply and delivery and militate against patient choice amongst vaccine brands and platforms. Second, the legitimate purposes for interference listed in Article 8(2) are readily aligned to the interferences with choice that seek to alleviate the global pandemic. They include ‘the interests of national security’ and ‘public safety or the economic well-being of the country’ which might be threatened by new variants if global inequity is not addressed, and ‘the protection of health’ which would be at risk if new waves of COVID-19 and potentially more deadly variants emerge. The Oviedo Convention on Human Rights and Biomedicine^132^ stresses in its preamble the need for international co-operation, and Article 3 requires equitable access to healthcare. UNESCO’s Universal Declaration, though a soft law instrument, recommends by Article 13 ‘Solidarity among human beings and international cooperation towards that end’. It goes on to say:

Article 24(3): States should respect and promote solidarity between and among States, as well as individuals, families, groups and communities, with special regard for those rendered vulnerable by disease or disability or other personal, societal or environmental conditions and those with the most limited resources.

In *Stoicescu v Romania* the court made clear:

It is not the Court’s task to substitute itself for the competent domestic authorities in determining the best policy to adopt in dealing with problems of public health and safety … In that connection it accepts that an impossible or disproportionate burden must not be imposed on the authorities without consideration being given in particular to the operational choices which they must make in terms of priorities and resources. This results from the wide margin of appreciations States enjoy.^133^

We have referred to the margin of appreciation in how states balance national interests (which we have argued should be alert to the global impact of decisions) and individual rights, we end by noting that provided a complaint is admissible, states must at least engage in a balancing exercise. In *Polat v Austria*,^134^ when a mother’s baby was born prematurely and died, the baby’s organs were removed notwithstanding her objections, in pursuit of public health aims. The lack of consideration of her position—the lack of any balancing exercise—breached Article 8. States may be advised, therefore, that the potential for a failure to protect choice to interfere with Article 8 does at least provide a reason to regularly reassess the balance as the national and global pandemic context shifts.

## VI. CONCLUSIONS

When COVID-19 vaccinations were first rolled out, the advantages of recipient choice amongst vaccination brands were outweighed by the limited value of choice given the lack of evidence about relative efficacy and risk, and utilitarian considerations favouring the swift, equitable and decisive national roll out of vaccinations to maximum numbers. As the impact of the virus continues and some countries transition to living with COVID-19, the emergency nature of pandemic-related policies abates and public expectations of choice—where choices are indeed available—are likely to rise. We have pointed to several factors that will increase pressure on some countries, including the UK, to offer greater choice if multiple clinically relevant alternatives are available in that country. Indeed, we recognise that choice might be highly valued by recipients and can have instrumental value including enhanced vaccine uptake.

These factors must be balanced with the impact of recipient choice on efficacy, cost and ease of vaccine supply and distribution. Even as COVID-19 strategy is de-escalated, the risk of new waves and variants of concern remains, and additional new, adapted and improved vaccines are likely to be authorised for some time to come. In such contexts, issues of global vaccine access and equity are also likely to remain or re-emerge. In the transition to normality, ‘normalisation’^135^ requires elements of COVID-19 strategy to be ingrained in policy in the longer-term. We have argued that an important policy consideration is the impact that choice in one state would have on global supply and distribution of vaccines. There can be no return to normal in a global pandemic until a global solution is found and it behoves us, both for this pandemic and future pandemics to come, that we learn to act collectively to achieve this.

## Footnotes

1 WHO, *COVID-19 Vaccine Tracker and Landscape* (3 May 2022) <https://www.who.int/publications/m/item/draft-landscape-of-covid-19-candidate-vaccines> accessed 22 August 2022.

2 H Petousis-Harris, ‘Assessing the Safety of COVID-19 Vaccines: A Primer’ (2020) 43(12) Drug Safety 1205.

3 J Holder, ‘Tracking Coronavirus Vaccinations Around the World’ *The New York Times* (updated 23 August 2022) <https://www.nytimes.com/interactive/2021/world/covid-vaccinations-tracker.html> accessed 23 August 2022.

4 H Ledford, D Cyranoski and R van Noorden, ‘The UK Has Approved a COVID Vaccine — Here’s What Scientists Now Want to Know’ (2020) Nature (3 December) <https://www.nature.com/articles/d41586-020-03441-8> accessed 6 May 2022.

5 See ‘Britain Sending Millions More COVID Doses to Developing Nations’ *Reuters* (30 October 2021) <https://www.reuters.com/world/uk/britain-sending-millions-more-covid-doses-developing-nations-2021-10-30/> accessed 6 May 2022.

6 See DHSC, *JCVI Advice on the UK Vaccine Response to the Omicron Variant* (29 November 2021).

7 NHS, Coronavirus (COVID-19) Vaccine <https://www.nhs.uk/conditions/coronavirus-covid-19/coronavirus-vaccination/coronavirus-vaccine/> accessed 6 May 2022.

8 Gov.UK, ‘Novavax COVID-19 Vaccine Nuvaxovid Approved by MHRA’ (3 February 2022) <https://www.gov.uk/government/news/novavax-covid-19-vaccine-nuvaxovid-approved-by-mhra> accessed 6 May 2022.

9 MHRA, ‘Valneva COVID-19 Vaccine approved by MHRA’ UK.Gov (14 April 2022) <https://www.gov.uk/government/news/valneva-covid-19-vaccine-approved-by-mhra> accessed 20 April 2022.

10 MHRA, ‘First Bivalent COVID-19 Booster Vaccine Approved by UK Medicines Regulator’ *UK.Gov* (15 August 2022) <https://www.gov.uk/government/news/first-bivalent-covid-19-booster-vaccine-approved-by-uk-medicines-regulator> accessed 15 August 2022.

11 A Standing Advisory Committee established under the NHS (Standing Advisory Committees) Order 1981 (SI 1981/597) to advise health departments on immunisations.

12 Importantly, in this context, we are focused on the limits imposed by states on vaccine recipient choice. However, in taking this focus we are not suggesting that states should not have discretion over what vaccine they provide to their populations at the state level, indeed, we regard ensuring state choice around vaccines for certain groups/populations based on clinical effectiveness as crucial.

13 European Medicines Agency*, COVID-19 Vaccines* (2022) <https://www.ema.europa.eu/en/human-regulatory/overview/public-health-threats/coronavirus-disease-covid-19/treatments-vaccines/covid-19-vaccines> accessed 22 August 2022.

14 Editorial, ‘COVID Is Here to Stay: Countries Must Decide How to Adapt’ Nature (10 January 2022) <https://www.nature.com/articles/d41586-022-00057-y> accessed 6 May 2022.

15 WHO (n 1).

16 S Tuvić, ‘How Vaccine Doubts Have Put the Handbrake on Serbia’s Rapid Rollout’ *Euronews* (3 August 2021) <https://www.euronews.com/2021/08/03/how-vaccine-doubts-have-put-the-handbrake-on-serbia-s-rapid-rollout> accessed 6 May 2022.

17 See, eg *Montgomery v Lanarkshire Health Board* [2015] UKSC 11, [69] and [87] (Lords Kerr and Reed).

18 We do not consider the scope for legal action if vaccinators contravene applicable clinical guidelines about contraindications based on the recipient’s age, history, or existing health conditions or well-trodden ground relating to whether a state should provide adequate medical care, fund expensive treatment that will limit national resources, or refund treatment abroad. Nor do we consider policies that allow some people choice and deny it to others, potentially exacerbating social inequalities in the process, on which see MT Hughes and others, ‘Opinion: The importance of Offering Vaccine Choice in the Fight against COVID-19’ (2021) 118(43) Proceedings of the National Academy of Sciences e2117185118.

19
*Tysiac v Poland*, App no 5410/03 (2007) [107]; *Pretty v UK* (2002) 35 EHRR 1, [64]–[67].

20
*Arskaya v Ukraine* App no 45076/05 (2013) [69].

21 In some countries (including some HIC), this has not been possible given shortfalls in supply: See I Torjesen, ‘Covid-19 Vaccine Shortages: What Is the Cause and What Are the Implications?’ (2021) 372 BMJ n781. Delays have also been experienced at a regional level– see ‘Coronavirus: EU Vaccine Woes Mount as New Delays Emerge’ *BBC News* (23 January 2021) <https://www.bbc.com/news/world-europe-55771223> accessed 6 May 2022.

22 See for example, WHO, ‘From the ‘New Normal’ to a ‘New Future’: A Sustainable Response to COVID-19’ (2020) <https://www.who.int/westernpacific/news-room/commentaries/detail-hq/from-the-new-normal-to-a-new-future-a-sustainable-response-to-covid-19> accessed 6 May 2022.

23 UK Health Security Agency, ‘Public reminded to stay safe as COVID-19 England restrictions lift’ (24 February 2022) <https://www.gov.uk/government/news/public-reminded-to-stay-safe-as-covid-19-england-restrictions-lift> accessed 6 May 2022.

24 See G Capano and others, ‘Long-Term Policy Impacts of the Coronavirus: Normalization, Adaptation, and Acceleration in the Post-COVID State’ (2022) 41(1) Policy and Society 1, 5.

25 UNDP, *Global Dashboard for Vaccine Equity* (2022) <https://data.undp.org/vaccine-equity/> accessed 28 February 2022.

26 See J Juno and A Wheatley, ‘Are New COVID Variants Like Omicron Linked to Low Vaccine Coverage? Here’s What the Science Says’ *The Conversation* (29 November 2021) <https://theconversation.com/are-new-covid-variants-like-omicron-linked-to-low-vaccine-coverage-heres-what-the-science-says-170262>; I Kottasová, ‘Vaccine Inequity and Hesitancy Made the Omicron Variant More Likely, Scientists Say’ *CNN News* (29 November 2021) <https://edition.cnn.com/2021/11/28/world/omicron-coronavirus-variant-vaccine-inequity-intl-cmd/index.html> both accessed 6 May 2022.

27 DHSC, ‘An Update on the Coronavirus Vaccine’ (2 December 2020) <https://www.gov.uk/government/speeches/an-update-on-the-coronavirus-vaccine-2-december-2020> accessed 6 May 2022.

28 See W Booth and K Adam, ‘Can I Choose My Covid Vaccine? Strong Opinions on Oxford vs Pfizer Emerge in UK’ *The Washington Post* (21 February 2021) <https://www.washingtonpost.com/world/europe/covid-vaccines-choice-pfizer-astrazeneca/2021/02/20/0beaceb0-5f2f-11eb-a177-7765f29a9524_story.html> accessed 20 August 2022.

29 FP Polack and others, ‘Safety and Efficacy of the BNT162b2 mRNA Covid-19 Vaccine’ (2020) 383(27) New England Journal of Medicine 2603.

30 Eg Scottish Government, *FOI Release: COVID-19 Vaccination Options within Scotland* (16 September 2021) FOI/202100240758 <https://www.gov.scot/publications/foi-202100240758/> accessed 6 May 2022.

31 MHRA (n 9).

32 MHRA (n 10).

33 C Wilson, ‘Why the UK Changed Covid-19 AstraZeneca Vaccine Advice for under-30s’ New Scientist (7 April 2021) <https://www.newscientist.com/article/2274018-why-the-uk-changed-covid-19-astrazeneca-vaccine-advice-for-under-30s/> accessed 6 May 2022.

34 See, for example, the links between the H1N1 2009 influenza vaccine, Pandemrix and narcolepsy: E Miller and others, ‘Risk of Narcolepsy in Children and Young People Receiving AS03 Adjuvanted Pandemic A/H1N1 2009 Influenza Vaccine: Retrospective Analysis’ (2013) 346 BMJ f794.

35 See, for example, L Taylor, ‘Covid-19: CoronaVac Immunity Is Strongest after Boosting with a Different Vaccine’ (2022) 376 BMJ o210.

36 DHSC, JCVI Statement, September 2021: COVID-19 Booster Vaccine Programme for Winter 2021 to 2022 (14 September 2021) <https://www.gov.uk/government/publications/jcvi-statement-september-2021-covid-19-booster-vaccine-programme-for-winter-2021-to-2022> accessed 6 May 2022.

37 Eg R Luscombe, ‘Springsteen’s Back on Broadway – And Now AstraZeneca Vaccine Recipients Are Allowed’ *The Guardian* (18 June 2021) <https://www.theguardian.com/music/2021/jun/18/bruce-springsteen-vaccine-astrazeneca-broadway> accessed 6 May 2022.

38 Heath Service Executive, Ireland, *Medicine Protocol for the Administration of Vaxzevria^®^ Vaccine (AstraZeneca) to Vaccine Recipients* (2021) <https://www.hse.ie/eng/health/immunisation/hcpinfo/covid19vaccineinfo4hps/astrazenecamedprot.pdf> accessed 3 December 2021.

39 S Pagaria, ‘Consumerism in Healthcare – Current Status, Benefits and Challenges’ (2020) 20 Health Management <https://healthmanagement.org> accessed 7 March 2022.

40
*Montgomery* [75] (Lords Kerr and Reed) (n 17). The role of consumerism in the health context, has been critiqued including, in: E Jackson, ‘Challenging the Comparison in *Montgomery* between Patients and ‘Consumers Exercising Choices’’ (2021) 29(4) Medical Law Review 595; TT Arvind and AM McMahon, ‘Responsiveness and the Role of Rights in Medical Law: Lessons from Montgomery’ (2020) 28 Medical Law Review 445.

41
*Montgomery* (n 17) [75] (Lords Reed and Kerr).

42 C El-Haddad, I Hegazi and W Hu, ‘Understanding Patient Expectations of Health Care: A Qualitative Study’ (2020) 7(6) Journal of Patient Expectations 1724.

43 E Karafillakis and others, ‘COVID-19 in Europe: New Challenges for Addressing Vaccine Hesitancy’ (2022) 399 Lancet P699; Editorial, ‘Vaccine Hesitancy Is Taking a Toll in Europe’s East’ *Financial Times* (15 December 2021) <https://www.ft.com/content/076c3c8e-deb8-4fe7-897b-6eab47a01163> accessed 6 May 2022; JI Steinert and others, ‘COVID-19 Vaccine Hesitancy in Eight European Countries: Prevalence, Determinants, and Heterogeneity’ (2022) 8(17) Science Advances doi:10.1126/sciadv.abm9825.

44 E Paul, A Steptoe and D Fancourt, ‘Attitudes towards Vaccines and Intention to Vaccinate Against COVID-19’ (2021) 1 Lancet Regional Health – Europe 100012.

45 On which see A Guibilini and others, ‘The ‘Ethical’ COVID-19 Vaccine Is the One that Preserves Lives: Religious and Moral Beliefs on the COVID-19 Vaccine’ (2021) 14(3) Public Health Ethics 242.

46 See Public Health England, *Vaccines and Porcine Gelatine* (2015) (updated 2020) <https://www.gov.uk/government/publications/vaccines-and-porcine-gelatine> accessed 6 May 2022.

47 P Sprengholz and others, ‘The Power of Choice: Experimental Evidence that Freedom to Choose a Vaccine against COVID-19 Improves Willingness to Be Vaccinated’ (2021) 87 European Journal of Internal Medicine 106. And see previous study: D Determann and others, ‘Acceptance of Vaccinations in Pandemic Outbreaks: A Discrete Choice Experiment’ PLoS One (24 July 2014) doi:10.1371/journal.pone.0102505.

48 Tuvić (n 16).

49 Hughes and others (n 18).

50 Gavi, ‘What Is the Novavax Vaccine, and Why Does the World Need Another Type of COVID-19 Vaccine?’ (14 February 2022) <https://www.gavi.org/vaccineswork/what-novavax-vaccine-and-why-does-world-need-another-type-covid-19-vaccine> accessed 6 May 2022.

51 App nos 47621/13, 3867/14, 73094/14, 19306/15, 19298/15, and 43883/15 (8 April 2021).

52 D Archard and others, ‘Compulsory Childhood Vaccination: Human Rights, Solidarity, and Best Interests’ (2021) 29(4) Medical Law Review 716.

53 See DB Kramer and others, ‘Choices in a Crisis — Individual Preferences among SARS-CoV-2 Vaccines’ (2021) 384 New England Journal of Medicine e62.

54 More recently, there have been more positive reports on therapeutics such as Paxlovid against mitigating serious risks posed by COVID-19. See NHS England, ‘Highest Risk COVID-19 Patients Receive ‘Brilliant’ New Antivirals at Home’ (9 April 2022) <https://www.england.nhs.uk/2022/04/highest-risk-covid-19-patients-receive-brilliant-new-antivirals-at-home/>. However, it has been reported that shortages of therapeutics for LMICs are likely: see A Green, ‘The Looming COVID-19 Treatment Equity Gap’ *Devex* (10 March 2022) <https://www.devex.com/news/the-looming-covid-19-treatment-equity-gap-102816> both accessed 6 May 2022.

55 JCVI, Advice on Priority Groups for COVID-19 Vaccination (30 December 2020) <https://www.gov.uk/government/publications/priority-groups-for-coronavirus-covid-19-vaccination-advice-from-the-jcvi-30-december-2020/joint-committee-on-vaccination-and-immunisation-advice-on-priority-groups-for-covid-19-vaccination-30-december-2020> accessed 6 May 2022.

56 ibid.

57 DHSC, Office for National Statistics, Government Actuary’s Department and Home Office, *Direct and Indirect Impacts of COVID-19 on Excess Deaths and Morbidity: Executive Summary* (15 July 2020) <https://assets.publishing.service.gov.uk/government/uploads/system/uploads/attachment_data/file/907616/s0650-direct-indirect-impacts-covid-19-excess-deaths-morbidity-sage-48.pdf> accessed 6 May 2022.

58 ibid.

59 The Human Medicines (Coronavirus and Influenza) (Amendment) Regulations 2020 (SI 2020/1125) amended the Human Medicines Regulations 2012.

60 In the US context, see C McClay, ‘Covid Vaccines: Why Some Americans Are Choosy about Their Jab’ *BBC News* <https://www.bbc.com/news/world-us-canada-56410179>; in Germany, see ‘Germans Clamor for Covid Vaccines, but Shun AstraZeneca’s Offering’ *NY Times* (25 February 2021) <https://www.nytimes.com/2021/02/25/world/europe/germany-coronavirus-vaccines-astrazeneca.html> both accessed 6 May 2022.

61 P Kelly, A Kirk and K Ahmed, ‘Covid Vaccine Figures Lay Bare Global Inequality as Global Target Missed’ *The Guardian* (21 July 2022) <https://www.theguardian.com/global-development/2022/jul/21/covid-vaccine-figures-lay-bare-global-inequality-as-global-target-missed> accessed 22 August 2022.

62 N Badshah, ‘UK to Face Delay in Delivery of Pfizer Covid Vaccine’ *The Guardian* (15 January 2021) <https://www.theguardian.com/world/2021/jan/15/uk-to-face-short-term-delay-in-delivery-of-pfizer-vaccine>; J Elgot and M Safi, ‘Matt Hancock Confirms Dip in UK Covid Vaccine Supply for April’ *The Guardian* (18 March 2021) <https://www.theguardian.com/world/2021/mar/18/matt-hancock-tells-mps-retest-on-covid-doses-and-delays-in-india-will-hit-uk-jabs> both accessed 6 May 2022.

63 Eg M Brazier and J Miola, ‘Bye Bye Bolam: A Medical Litigation Revolution? (2000) 8 Medical Law Review 85, 112; M Brazier, ‘Do No Harm—Do Patients Have Responsibilities Too?’ (2006) 65 Cambridge Law Journal 397; J Coggon and J Miola, ‘Autonomy, Liberty, and Medical Decision-Making’ (2011) 70 Cambridge Law Journal 523; J Montgomery, ‘Patient No Longer? What Next in Healthcare Law?’ (2017) 70 Current Legal Problem 73; E Cave and C Milo, ‘Informing Patients: The Bolam Legacy’ (2020) 20(2) Medical Law International 103.

64 Cave and Milo (n 63) 118.

65 Arvind and McMahon (n 40).

66 ibid 452, citing B McKinstry, ‘Do Patients Wish to Be Involved in Decision Making in the Consultation? A Cross Sectional Survey with Video Vignettes’ (2000) 321 BMJ 867; S Joffe and others, ‘What Do Patients Value in Their Hospital Care? An Empirical Perspective on Autonomy Centred Bioethics’ (2003) 29 Journal of Medical Ethics 103, 106.

67 GN Samuel and others, ‘Healthcare Professionals’ and Patients’ Perspectives on Consent to Clinical Genetic Testing: Moving Towards a More Relational Approach’ (2017) 18 BMC Medical Ethics 47; Arvind and McMahon (n 40) 454.

68 See BA Brody, ‘Making Informed Consent Meaningful’ (2001) 23 IRB Ethics & Human Research 1; Arvind and McMahon (n 40) 454.

69 Arvind and McMahon (n 40) 460.

70 Community Research, *Doctors’ Attitudes to Consent and Shared Decision Making: Full Research Report for the GMC*, (June 2017).

71 See H Ledford, ‘Why COVID Vaccines Are So Difficult to Compare’ Nature (23 February 2021) <https://www.nature.com/articles/d41586-021-00409-0>; W Shi Lee and H Xhi Tan, ‘Which COVID Vaccine Is Best? Here’s Why That’s Really Hard to Answer’ *The Conversation* (16 June 2021) <https://theconversation.com/which-covid-vaccine-is-best-heres-why-thats-really-hard-to-answer-161185> accessed 6 May 2022.

72 K Katella, ‘Comparing the COVID-19 Vaccines: How Are They Different?’ Yale Medicine (3 November 2021) <https://www.yalemedicine.org/news/covid-19-vaccine-comparison> accessed 6 May 2022.

73 Eg M Gollwitzer and others, ‘Public Acceptance of Covid-19 Lockdown Scenarios’ (2021) 56(4) International Journal of Psychology 551.

74 UK Health Security Agency (n 23).

75 Cabinet Office, *COVID-19 Response: Living with COVID* (23 February 2022) <https://www.gov.uk/government/publications/covid-19-response-living-with-covid-19/covid-19-response-living-with-covid-19> accessed 6 May 2022.

76 ibid.

77 R McKie, ‘Next Covid Strain Could Kill Many More, Warn Scientists ahead of England Restrictions Ending’ *The Guardian* (12 February 2022) <https://www.theguardian.com/world/2022/feb/12/scientists-plead-caution-covid-restrictions-lifted-england> accessed 6 May 2022. We do not comment here on the justifications for the English approach to de-escalation, which suggests a binary view of liberty or restrictions. See: J Coggon, ‘Personal Responsibility versus Legal Obligation? Why Simplistic Binaries Make for Bad Pandemic Responses’ *Nuffield Council on Bioethics Blogpost* (12 July 2021) <https://www.nuffieldbioethics.org/blog/personal-responsibility-versus-legal-obligation-why-simplistic-binaries-make-for-bad-pandemic-responses> accessed 6 May 2022.

78 On the likelihood of climate change sparking future pandemics, see: N Gilbert, ‘Climate Change Will Force New Animal Encounters — And Boost Viral Outbreaks’ (2022) 605 Nature 20.

79 A Katzourakis, ‘COVID-19: Endemic Doesn’t Mean Harmless’ (2022) 601 Nature 485.

80 See for example (in the context of the USA), MT Hughes and others, ‘The Importance of Offering Vaccine Choice in the Fight against COVID-19’ (2021) 118(43) Proceedings of the National Academy of Sciences e2117185118.

81 See J Coggon, *What Makes Health Pubic?* (CUP 2012) xiii.

82
*Re H (A Child) (Parental Responsibility: Vaccination)* [2020] EWCA Civ 664 [84].

83
*Montgomery* (n 17).

84 ibid [77]–[78] (Lords Kerr and Reed).

85 ibid [90] (Lords Reed and Kerr), [110], [116] (Lady Hale).

86 ibid [80] (Lords Reed and Kerr), [108] (Lady Hale).

87 ibid [81] (Lords Reed and Kerr).

88 See, eg A Potappel and others, ‘Patient Choice of a Healthcare Provider at the Point of Referral’ (2018) 28(4) European Journal of Public Health cky213.426.

89 See The Lancet Infectious Diseases, ‘COVID-19 Vaccine Equity and Booster Doses’ (2021) 21(9) Lancet Infectious Disease 1193: ‘Vulnerable people in HICs have already been prioritised; vulnerable people in LMICs cannot wait until 2023 for their turn, and this wait is in the best interest of no one.’

90 DP Mancini and M Stabe, ‘Covid Boosters in Rich Countries Outnumber All Jabs among Poor Nations’ *The Financial Times* (19 December 2021) <https://www.ft.com/content/5144cd19-2c67-46d0-a37d-a869006bfbdb> accessed 25 April 2021.

91 UNDP (n 25).

92 ibid.

93 ‘Science Uncertain about Need for Vaccine Boosters, WHO Says’ *Irish Times* (18 August 2021) <https://www.irishtimes.com/news/health/science-uncertain-about-need-for-vaccine-boosters-who-says-1.4650435> accessed 6 May 2022.

94 EM Choi, ‘COVID-19 Vaccines for Low- and Middle-Income Countries’ (2021) 115(5) Transactions of the Royal Society of Tropical Medicine Hygiene 447.

95 N Hassoun, ‘Against Vaccine Nationalism’ (2021) 47 Journal of Medical Ethics 773.

96 K Ferguson and A Caplan, ‘Love Thy Neighbour? Allocating Vaccines in a World of Competing Obligations’ (2021) 47 Journal of Medical Ethics e20.

97 ET Tagoe and others, ‘COVID-19 Vaccination in Lower-Middle Income Countries: National Stakeholder Views on Challenges, Barriers, and Potential Solutions’ (2021) 9 Frontiers in Public Health doi:10.3389/fpubh.2021.709127.

98 A Maxmen, ‘The Fight to Manufacture COVID Vaccines in Lower-Income Countries’ Nature (15 September 2021) <https://www.nature.com/articles/d41586-021-02383-z> accessed 6 May 2022.

99 L Barnéoud, ‘The Huge Waste of Expired Covid-19 Vaccines’ *Le Monde* (4 April 2022) <https://www.lemonde.fr/en/science/article/2022/04/04/the-huge-waste-of-expired-covid-19-vaccines_5979632_10.html> accessed 6 May 2022.

100 But see JS Solís Arce and others, ‘COVID-19 Vaccine Acceptance and Hesitancy in Low- and Middle-Income Countries’ (2021) 27 Nature Medicine 1385.

101 For example, the COVAX Facility. See Gavi, ‘Gavi and Global Health Actors Collaborate to Accelerate COVID-19 Technologies for All’ (24 April 2020) <https://www.gavi.org/news/media-room/gavi-and-global-health-actors-collaborate-accelerate-covid-19-technologies-all> accessed 6 May 2022.

102 Hansard, ‘Covid-19: Vaccinations and Global Public Health’ HL deb 9 September 2021, vol 814, col 1025.

103 ibid.

104 ‘WHO Director-General's Opening Remarks at the Special Ministerial Meeting: A Vaccine for All’ (16 April 2021) <https://www.who.int/director-general/speeches/detail/who-director-general-s-opening-remarks-at-the-special-ministerial-meeting-a-vaccine-for-all> accessed 6 May 2022.

105 See discussion in: S Geiger and A McMahon, ‘Analysis of the Institutional Landscape and Proliferation of Proposals for Global Vaccine Equity for COVID-19: Too Many Cooks or Too Many Recipes?’ (2021) Journal of Medical Ethics. doi:10.1136/medethics-2021-107684.

106 ibid.

107 S Thambisetty and others, ‘The TRIPS Intellectual Property Waiver Proposal: Creating the Right Incentives in Patent Law and Politics to End the COVID-19 Pandemic’ (24 May 2021). LSE Legal Studies Working Paper No 06/2021 <https://ssrn.com/abstract=3851737> or <http://dx.doi.org/10.2139/ssrn.3851737> accessed 23 August 2022.

108 For the proposal, see <http://freepdfhosting.com/4d79fc6c70.pdf>; A Green, ‘TRIPS Waiver Compromise Draws Mixed Response’ *Devex* (17 March 2022) <https://www.devex.com/news/trips-waiver-compromise-draws-mixed-response-102860> accessed 22 April 2022.

109 A Green, 'WTO Finally Agrees on a TRIPS Deal. But Not Everyone Is Happy’ *Devex* (17 June 2022) <https://www.devex.com/news/wto-finally-agrees-on-a-trips-deal-but-not-everyone-is-happy-103476> accessed 28 June 2022.

110 See WHO, *Vaccine Equity* <https://www.who.int/campaigns/vaccine-equity> accessed 22 August 2022.

111 WHO, *Global Covid-19 Vaccination Strategy in a Changing World: July 2022 Update* (WHO 2022) 3.

112 As, for example, in *Gardner & Harris v Secretary of State for Health and Social Care & Ors* [2022] EWHC 967 (Admin).

113 UNESCO, *Universal Declaration on Bioethics and Human Rights* (2005).

114 See for example *VC v Slovakia* App no 18968/07 ECHR 1888 (2011) [86]; *Herrmann v Germany* App no 9300/07 (2012) [23]; *Nedescu v Romania* App no 70035/10 (2018) [46].

115
*Centre For Legal Resources on Behalf of Valentin Câmpeanu v Romania* App no 47848/08, GC (2014); *Mehmet Sentürk and Bekir Sentürk v Turkey* App no 13423/09 (2013) 60 EHRR 4; *Asiye Genc v Turquie* App no 24109/07 (27 January 2015).

116
*Aggerholm v Denmark*, App no 45439/18 [2020] ECHR 628.

117 See for example *Polat v Austria*, App no 12886/16 (2021) [89]–[91].

118 An analogous case from the European Court of Justice is *A v Veselības Ministrija* [2020] ECJ C-243/19 which recognised the importance of patient choice when a father sought state funding for alternative treatment elsewhere in the EU for his son, that would comply with the family’s religious convictions. The Opinion takes into consideration the likely impact of disruption to state planning as a justification for restrictions on patient choice.

119
*Powell and Rayner v UK* [1990] 12 EHRR 355, [1990] ECHR 2, [41]: ‘Whether the present case be analysed in terms of a positive duty on the State to take reasonable and appropriate measures to secure the applicants' rights under paragraph 1 of Article 8 (art. 8-1) or in terms of an “interference by a public authority” to be justified in accordance with paragraph 2 (art. 8-2), the applicable principles are broadly similar. In both contexts regard must be had to the fair balance that has to be struck between the competing interests of the individual and of the community as a whole; and in both contexts the State enjoys a certain margin of appreciation in determining the steps to be taken to ensure compliance with the Convention.’

120
*X and Y v The Netherlands* (1985) 8 EHRR 235, [1985] ECHR 4 [22]; *Vavřička and Others v the Czech Republic* (n 51) [261].

121
*A, B and C v Ireland* [GC], App no 25579/05 (16 December 2010) para 247.

122
*X, Y and Z v UK*, App no 21830/93 [1997] ECHR 20. The margin is particularly wide if the claim invokes sensitive moral issues.

123
*Ladygin v Russia*, App no 35365/05 (20 August 2011).

124 This can include important points of principle for the applicant: eg a prohibition on growing a beard in prison that caused mental suffering was not insignificant: *Biržietis v Lithuania*, App no 49304/09 (2016).

125 App no 52806/09 [244].

126
*Glass v UK* (2004) EHRR 341 and see *Tysiac v Poland* (n 19).

127
*Valentina Pentiacova and Others against Moldova* App no 14462/03 (2005).

128
*Sheffield and Horsham v UK* (1998) 27 EHRR 163 and see ibid.

129 [2011] ECHR 1193 (26 July 2011).

130 ibid [34].

131 ibid [55]–[63].

132 Council of Europe, *Convention for the Protection of Human Rights and Dignity of the Human Being with regard to the Application of Biology and Medicine: Convention on Human Rights and Biomedicine* (ETS No 164) (Oviedo, 1997). It is not signed and ratified by all Members of the Council of Europe, eg Germany and the UK.

133 [2011] ECHR 1193 (26 July 2011) [59]. And see *Osman v UK* [GC] App no 23452/94 (28 October 1998) [116].

134 App no 12886/16 (2021) [89]–[91].

135 Capano and others (n 24).

